# Bleeding Efficiency, Microbiological Quality and Oxidative Stability of Meat from Goats Subjected to Slaughter without Stunning in Comparison with Different Methods of Pre-Slaughter Electrical Stunning

**DOI:** 10.1371/journal.pone.0152661

**Published:** 2016-04-01

**Authors:** Azad Behnan Sabow, Idrus Zulkifli, Yong Meng Goh, Mohd Zainal Abidin Ab Kadir, Ubedullah Kaka, Jurhamid Columbres Imlan, Ahmed Abubakar Abubakar, Kazeem Dauda Adeyemi, Awis Qurni Sazili

**Affiliations:** 1 Department of Animal Science, Faculty of Agriculture, Universiti Putra Malaysia, 43400 UPM Serdang, Selangor, Malaysia; 2 Institute of Tropical Agriculture, Universiti Putra Malaysia, 43400 UPM Serdang, Selangor, Malaysia; 3 Department of Veterinary Preclinical Sciences, Universiti Putra Malaysia, 43400 UPM Serdang, Selangor, Malaysia; 4 Department of Veterinary Clinical Studies, Faculty of Veterinary Medicine, Universiti Putra Malaysia, 43400 UPM Serdang, Selangor, Malaysia; 5 Department of Electrical and Electronic Engineering, Faculty of Engineering, Universiti Putra Malaysia, 43400 UPM Serdang, Selangor, Malaysia; 6 Halal Products Research Institute, Universiti Putra Malaysia, 43400 UPM Serdang, Selangor, Malaysia; 7 Centre for Electromagnetic and Lighting Protection Research, Universiti Putra Malaysia, 43400 UPM Serdang, Selangor, Malaysia; 8 Department of Animal Resource, University of Salahaddin, Erbil, Kurdistan Region, Iraq; 9 Department of Veterinary Surgery and Obstetrics, Faculty of Animal Husbandry and Veterinary Sciences, Sindh Agriculture University Tandojam, Sindh, Pakistan; 10 Department of Animal Production, University of Ilorin, Ilorin, Nigeria; University of Florida, UNITED STATES

## Abstract

The influence of pre-slaughter electrical stunning techniques and slaughter without stunning on bleeding efficiency and shelf life of chevon during a 14 d postmortem aging were assessed. Thirty two Boer crossbred bucks were randomly assigned to four slaughtering techniques viz slaughter without stunning (SWS), low frequency head-only electrical stunning (LFHO; 1 A for 3 s at a frequency of 50 Hz), low frequency head-to-back electrical stunning (LFHB; 1 A for 3 s at a frequency of 50 Hz) and high frequency head-to-back electrical stunning (HFHB; 1 A for 3 s at a frequency of 850 Hz). The SWS, LFHO and HFHB goats had higher (p<0.05) blood loss and lower residual hemoglobin in muscle compared to LFHB. The LFHB meat had higher (p<0.05) TBARS value than other treatments on d 7 and 14 d postmortem. Slaughtering methods had no effect on protein oxidation. Higher bacterial counts were observed in LFHB meat compared to those from SWS, LFHO and HFHB after 3 d postmortem. Results indicate that the low bleed-out in LFHB lowered the lipid oxidative stability and microbiological quality of chevon during aging.

## Introduction

Consumers place much importance on food quality and safety at the point of purchase. Lipid and protein oxidation and microbial proliferation are limiting factors that influence safety and shelf life of meat [1]. During aging, the conversion of muscle to meat occurs along with quantitative transformations in many metabolites [2]. Consequently, meat becomes spoiled and unfit for consumption [3]. It has been reported that microbial levels between 6 to 7 log CFU cm^-2^ or g^-1^ during refrigerated storage are critical levels for meat spoilage [4]. In addition to microbial spoilage, lipid and protein oxidation are also regarded as the main cause of sensory, functional and nutritional quality deterioration in meat [5]. Lipid oxidation changes lead to unpleasant odor and taste, color deteriorations, degradation in proteins, and decline in shelf life, which could affect consumers’ health [6]. The chemical transformations that take place during protein oxidation are responsible for several biological modifications that affect meat quality, such as the solubility of proteins and protein fragmentation and aggregation [7,8].

In the livestock production and processing chain, the slaughtering of animals purposely for food (meat) is a delicate procedure governed by strict regulations related to food safety and hygiene, working conditions and animal welfare [9]. Traditionally, slaughter practices have dealt with factors, such as the type of stunning, that affect wholesomeness and quality of meat through the quantity of blood left within the carcass after bleeding. Bleeding efficiency is an essential requirement of slaughter procedures in order to obtain a high quality product. Blood loss is a major concern for meat processors as residual blood in the carcass is often related to a decrease in shelf life and meaty flavor. The high nutritive value, favorable pH, temperature, relative humidity and water activity makes blood an ideal medium for microbial proliferation [10]. The myoglobin and hemoglobin catalyze lipid and protein oxidation directly [11,12]. Thus, optimizing blood loss at slaughter to ensure product quality, promote shelf and reduce meat and carcass defects is a major concern of the meat processing industry [13,14].

In most Western countries, the slaughtering of livestock is governed by laws that require animals to be rendered insensible to pain before being shackled, hoisted and exsanguinated [15]. Nonetheless, in respect for human rights and freedom of worship, some exceptions have been given to slaughtering in line with the requirements of any religious faith that recommends a mode of slaughter whereby animals die by cutting the jugular veins and carotid arteries with a sharp instrument [15]. Because of the global population of Muslims and their interest in adhering to their religious doctrines, there is a robust market for meat obtained from animals slaughtered by the halal method [16,17]. Most non-Muslim consumers based their meat purchase decisions on the perception of safety and healthiness and sensory properties. Contrarily, Muslims consider the halal status of the meat before any other factors [18]. For Muslims, meat produced in the halal way is considered to be of the highest spiritual and ethical quality. However, halal slaughter is considered cruel and contrary to scientific wisdom of those who think that the animal must first be stunned prior to being slaughtered in order not to compromise the welfare of the animal [19]. These groups advocate for food labeling which identifies meat from animals slaughtered without stunning and they consider meat from such animals to be ideologically of the lowest quality [19]. Moreover, meat processors prefer slaughter methods that yield the best quality meat and thus, this has created a lot of debate among religious leaders, animal welfare officials and meat processors. Thus, the objective of this study was to examine the effect of different methods of electrical stunning in comparison with slaughter without stunning on bleeding efficiency and shelf life of goat meat.

## Materials and Methods

### Animal welfare

The experimental protocol was approved by the institutional animal care and use committee (IACUC) of the Universiti Putra Malaysia (Approval no. R052/2015).

### Animals, stunning and slaughter procedure

Thirty-two Boer crossbred bucks (8–10 months old; average body weight of 29.956 ± 1.587 kg) reared under similar management system, were purchased from a commercial goat farm. Slaughtering of animals with or without stunning was performed under minimal anesthesia. The minimal anesthesia required to ensure that a fixed amount of electrical current is delivered. This is crucial, as the incidence of mis-stunning is common among conscious animals [20]. Mis-stunning not only cause unintended tissue damage and welfare issues, but could result in higher number of animals required for the trial. Our prior studies [11,21], have also shown that there were no significant differences in terms of bleeding efficiency, physicochemical characteristics and shelf life between fully conscious and minimally anesthetized goats. The minimal anesthesia protocol used was in accordance with the procedures of Kaka et al. [22] and Gibson et al. [23]. Administration of anesthesia was induced by rapid injection of 5mg/ kg propofol through the cephalic vein and maintained with halothane in 100% oxygen. The goats were randomly allotted into four groups of eight animals each and subjected to either slaughter without stunning (SWS), low frequency head-only (LFHO), low frequency head-to-back (LFHB) or high frequency head-to-back (HFHB) pre-slaughter electrical stunning. The slaughtering protocol was carried-out at the Research Abattoir of Animal Science Department, Faculty of Agriculture, Universiti Putra Malaysia.

In the first group, the goats were slaughtered without stunning (SWS). The second group were stunned electrically by low frequency head-only method (LFHO) at a constant current of 1.0 A at 50 Hz for 3 s. The third group were electrically stunned with low frequency head-to-back method (LFHB) at a constant current of 1.0 A at 50 Hz for 3 s. In the last group, goats were electrically stunned by high frequency head-to-back method (HFHB) at a constant current of 1.0 A at 850 Hz for 3 s. Voltage was automatically adjusted according to the impedance of the animal’s head tissues. The stun was applied using electrical stunning transformer type CS-1 manufactured by Karl Schermer (Ettlingen, Baden-Württemberg, Germany). The stunning system was connected either through double-electrode scissor-type dry stunning tongs (Z3, Karl Schermer, Ettlingen, Germany) or triple-electrode, with an integral water spray, tongs (2A Handset, Kentmaster, Hartlebury, United Kingdom) to apply LFHO, LFHB and HFHB electrical stunning, respectively. The LFHO electrical stunning system comes together with a gun grip which is made of plastic insulating stainless steel electrodes, While the LFHB and HFHB electrical stunning system consist of two frontal stainless steel tongs and a third caudal (35cm) stainless steel plate electrode. The head electrodes are placed between the eyes and the ears on both sides for LFHO, while for LFHB and HFHB electrical stunning techniques, the head electrodes are placed behind the ears and the third electrode is placed on the vertebral column behind the position of the heart. The animals in the four treatments were humanely slaughtered following the guidelines outlined by the Department of Standards Malaysia [24] for halal slaughter practices.

### Muscle sampling and postmortem storage

After evisceration and carcass dressing, the left *Longissimus lumborum* (LL) between the 6^th^ and 8^th^ lumbar vertebra was dissected and divided into two portions, snap frozen in liquid nitrogen and stored at -80°C. The first portion was assigned for subsequent determination of thiobarbituric acid reactive substances (TBARS) at d 0. The second portion was assigned for subsequent determination of protein oxidation at d 0. The right *Semitendinosus* (ST) muscle was aseptically divided into two portions. The first portion was used for microbiological analysis at d 0 while the other was packaged in stomacher bags and stored in the chiller (4°C) for microbiological analysis at 1, 3, 7 and 14 d postmortem. The carcasses were placed in the chiller (4°C) and subsequent sampling was done at particular postmortem aging periods. The left LL muscle from the 9^th^ to 12^th^ lumbar vertebra was dissected into two parts. From the 9^th^ to 10^th^ lumbar vertebra were dissected into four portions at specific periods of 1, 3, 7 and 14 d postmortem for subsequent analysis of TBARS. The portion from the 11^th^ to 12^th^ lumbar vertebra was further dissected at 1, 3, 7 and 14 d postmortem for determination of protein oxidation. After, 1, 3, 7 and 14 d postmortem, muscle chops of about 3 cm thick dissected from the muscles and stored at -80°C until further analyses.

### Determination of blood loss

The animals were weighed before the treatment. During exsanguination, blood was collected in a plastic container and weighed. The amount of blood loss was measured using the following formula: Blood loss (%) = BW/ LW × 100 where: BW (kg) = weight of blood once the animal is dead (based on ECG) and LW (kg) = live body weight pre-slaughter.

### Hemoglobin quantification

Hemoglobin in the LL muscles were extracted and quantified following the procedure of O'Brien et al. [25] and described by Sabow et al. [11].

### Determination of lipid oxidation

Lipid oxidation was measured as 2-thiobarbituric acid reactive substances (TBARS) using QuantiChromTM TBARS Assay Kit (DTBA-100, BioAssay Systems, USA) following the procedure of Sabow et al. [11].

### Extraction of myofibrillar proteins

Myofibril proteins were isolated in accordance with the method of Morzel et al. [26] with slight modification as outlined by Nakyinsige et al. [27]. Approximately 2.5 g of pulverized muscle tissue was homogenized for 30 s on ice in 25 ml of extraction buffer containing 25 mM KCl, 150 mM NaCl, 4 mM EDTA, and 3 mM MgCl_2_ at pH 6.5 to which protease inhibitor (CALBIOCHEM®, Cat # 55140, EMD Bioscience, Inc. Germany) was added. After filtration, the homogenate was incubated at 4°C with stirring. This was followed by centrifugation at 2000 g for 15 min at 4°C using an Avanti^®^ J-26XPI centrifuge (BECKMAN COULTER®, USA). The pellets were washed twice with 25 ml of a 50 mM KCl solution at a pH of 6.4 and once with 25 ml of 20 mM phosphate buffer of pH 6. The pellets were finally re-suspended in the same phosphate buffer and the protein concentration of the samples was estimated in accordance to the Bradford method [28] with the aid of Protein Assay Kit II 500–0002 (Bio-Rad, USA) following the micro plate protocol for colorimetric procedure.

### Determination of free thiol content

The free thiol content was determined in accordance with Ellman’s technique of using 2, 2-dithiobis (5-nitropyridine) DTNP [29] with slight modifications as outlined by Morzel et al. [26]. Washed pellets containing 4 mg of myofibrillar proteins were dissolved in 3 ml of 100 mM phosphate buffer of pH 8 containing 8 M urea. About 30 μl of 10 mM DTNP (stock solution in ethanol) was added, followed by incubation for an hour at room temperature. The absorbance at 386 nm was measured using a spectrophotometer (Spectra SC Spectrophotometer, USA) against a blank buffer without protein. The absorbance of the blank was subtracted, and thiol concentration was estimated using an absorption coefficient of 14 mM^-1^ cm^-1^. The results were expressed in nanomoles of free thiol per milligram of protein.

### Determination of protein carbonyls

Carbonyl groups were estimated using Protein Carbonyl Colorimetric Assay Kit cat # 10005020 (Cayman, USA) following the manufacturer’s instructions. The carbonyl content was expressed as nanomoles of DNPH per milligram of protein.

### SDS-PAGE

**Fig 1:**
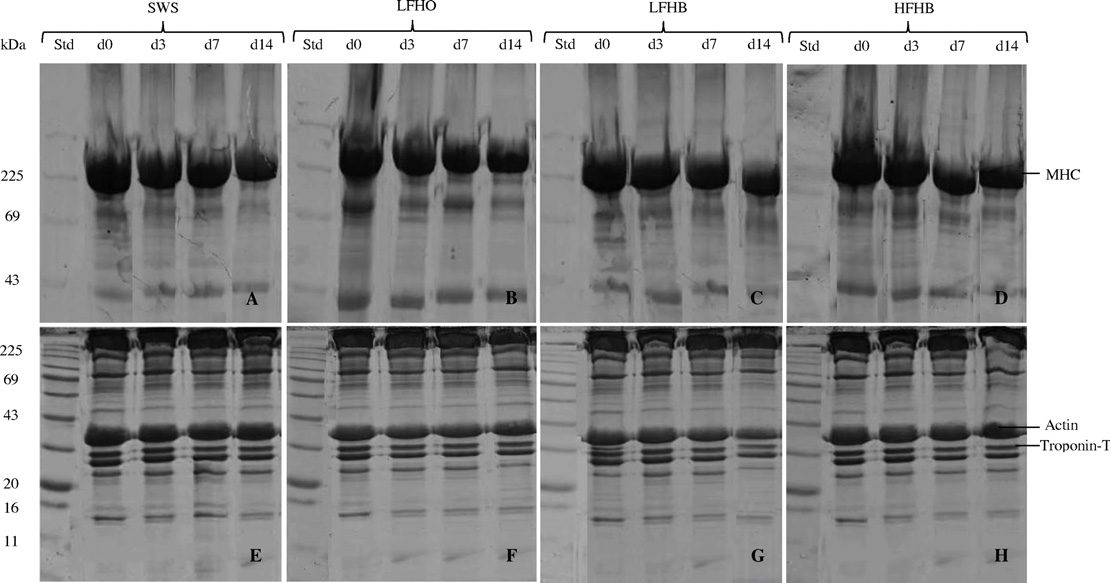
Representative SDS-PAGE showing myosin heavy chain (MHC), actin and troponin-T bands of *longissimus lumborum* muscle during postmortem aging periods in goats subjected to slaughter without stunning and slaughter following different methods of electrical stunning. Equal amount of protein (20 μg) of each sample was loaded and electrophoresed on a separate 5% (A, B, C, D) and 12% (E, F, G, H) SDS-PAGE under 120 V of constant voltage for about 90 min. The gels were then stained with coomassie blue staining for 60 min and destained with destaining solution for 45 min. The bands of MHC, actin and troponin-T were visualized using GS-800 Calibrated Imaging Densitometer. SWS: slaughter without stunning, LFHO: low frequency head-only, electrical stunning, LFHB: low frequency head-to-back electrical stunning, HFHB: high frequency head-to-back electrical stunning.

Myofibrillar proteins were mixed with sample buffer containing 62.5 mM Tris–HCl (pH 6.8), 5% (v/v) mercaptoethanol, 30% (v/v) glycerol, 2.3% (w/v) SDS, and 0.05% (w/v) bromophenol blue in a 1: 1 ratio and incubated at 90°C for 10 min. One dimensional SDS-PAGE was performed using polyacrylamide gels of 8 cm × 5.5 cm (length × width) and 0.8 mm thickness [30]. A 5% resolving gels were prepared for myosin heavy chain whereas a 12% resolving gels were prepared for actin and troponin-T. The resolving gels were over-layered with 4% stacking gel solution and kept overnight at 4°C in order to polymerize. A volume of 5 μl protein ladder (Blueye Prestained Protein Ladder; Cat No: PM007-0500 from GeneDirex^®^, Canada) was loaded into the first well, while an equivalent of 20 μg proteins of each sample were loaded into the remaining wells. Proteins were separated in running buffer (0.025 M Tris base, 0.192 M glycine, 0.1 SDS at pH 8.3) under constant voltage of 120 V and 400 mA for 90 min, in which the tracking dye reached the bottom of the gel. The gels were subsequently stained with staining solution (0.05% coomassie blue, 5% acetic acid and 15% methanol) for 60 min and destained with destaining solution (10% acetic acid and 30% methanol) for 30 min. A GS-800 Calibrated Imaging Densitometer (Bio-Rad, USA) was used to visualize the bands of myofibrillar proteins (Fig 1).

### Western blotting

The fractionated proteins that were initially separated from the samples based on their molecular weight through gel electrophoresis were then transferred from the gel onto polyvinylidene difluoride (PVDF) membranes using Trans-Blot^®^ SD semi-dry transfer system cell (Bio-Rad, USA). Myosin heavy chain was transferred at constant amperage of 250 mA per gel, voltage limit of 25 V for 135 min whereas actin was transferred at the same amperage and voltage for 45 min. The membranes were immersed in ponceau staining solution (0.5% ponceau S and 5% trichloroacetic acid) for 5 min to visualize the proteins of interest and to verify the electrophoretic transfer. The membranes were then washed with adequate deionized water, followed by one TBST buffer (100 mM Tris-HCl, 150 mM NaCl and 0.05% Tween 20) wash before being blocked with blocking buffer (5% BSA in TBST buffer) for 3 h at room temperature with constant shaking (Multi Shaker-FMS3-FINEPCR, Korea). For myosin heavy chain, the membranes were incubated overnight with 1: 500 dilution of primary antibody [Monoclonal Anti-Myosin (Skeletal, Fast), produced in mouse; Cat #. M4276 from Sigma- Aldrich, USA and Monoclonal Anti-Myosin (Skeletal, Slow), antibody produced in mouse; Cat # M842 from Sigma- Aldrich, USA in 3% BSA in TBST buffer]. Anti actin antibody produced in mouse; Cat # A4700 from Sigma- Aldrich, USA and monoclonal anti-troponin T, antibody produced in mouse; Cat # T6277 from Sigma- Aldrich, USA were the primary antibody used for actin and troponin-T, respectively. Subsequently, the membranes were washed three times in TBST buffer (5 min incubation at room temperature, with constant shaking). The membranes were further incubated at room temperature in 1:10000 dilution of secondary antibody [anti- mouse IgG (whole molecule)-peroxidase, antibody developed in rabbit; Cat # A9044 from Sigma- Aldrich, USA in 3% BSA in TBS-T buffer for 90 min]. This was followed by 3 times washing with TBST buffer. The blocked membranes were detected using a DAB substrate kit Code: E733, DAB SUBSTRATE SYSTEM (aMReSCO^®^, Ohio). Myosin heavy chain, actin and troponin-T band intensities were measured by Quantity one^®^ software on GS-800 Calibrated Imaging Densitometer (Bio-Rad, USA).

### Microbiological analysis

At 0, 1, 3, 7 and 14 d postmortem, 5 g of meat samples from ST muscle was drawn aseptically and transferred to a stomacher bag containing 45 ml of sterile 1.5% peptone water solution (Oxiod, England). The sample was homogenized using a stomacher (Inter Science, Saint-Nom-la-Bretèche, France) for 2 min at ambient temperature. In order to determine the microbial counts, 0.1 ml samples of serial dilutions (1:10 diluent, and peptone water) of meat homogenates were spread on the surface of dry media. Ten-fold dilutions were spread on petri dishes in duplicate for enumerations of total aerobic counts (TAC) on Plate Count Agar (Merck KGaA, Darmstadt, Germany) and lactic acid bacteria on Man, Rogosa and Sharpe agar (Merk KGaA, Darmstadt, Germany) following 3 d incubation at 32°C [1]. P*seudomonas* spp. counts were determined after 3 d incubation at 25°C on Centrimide Agar (Merck KGaA, Darmstadt, Germany) [1]. *Enterobacteriaceae* were enumerated after 24 h incubation on 3M™ Petrifilm™ Enterobacteriaceae Count Plates (3M Food Safety, USA) at 35°C following the manufacturer’s instructions. A colony counter (Stuart®; Burlington, VT, USA) was used for counting. The data (growth counts) were transformed to log10 values.

### Statistical analysis

The trial followed a completely randomized design. Data obtained were subjected to the GLM procedure of SAS (Statistical Analysis System, SAS Institute Inc., Cary, NC, USA). Mean were separated by Duncan multiple range test when the F test proved significant at p<0.05.

## Results and Discussion

The current study utilized minimal anesthetized animals, which is not an allowed practice for animals slaughtered for human consumption. Therefore, results should be interpreted taking into consideration potential physical and chemical traits of meat quality as well as other changes associated with anesthesia. It should be noted that differences between conscious and minimally anesthetized animals were mostly negligible based on our prior findings in physicochemical traits, residual blood content, oxidative stability and microbiological quality of meat [11, 21].

### Blood loss

**Fig 2:**
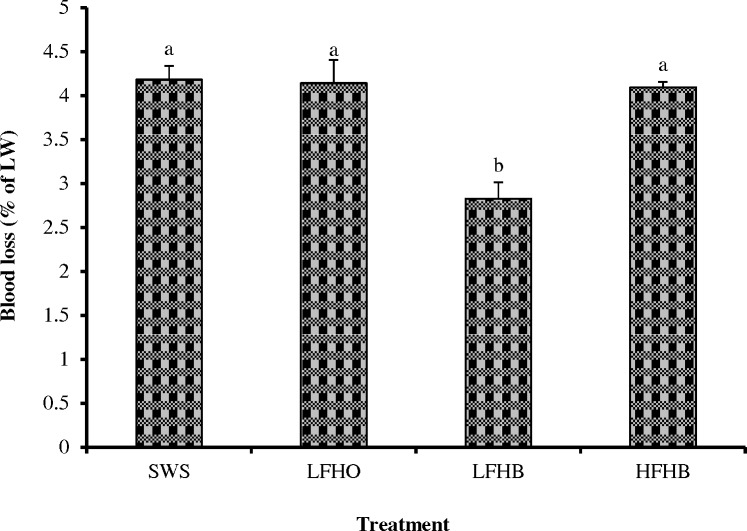
Blood loss in goats subjected to slaughter without stunning and slaughter following different methods of electrical stunning. SWS: slaughter without stunning, LFHO: low frequency head-only, electrical stunning, LFHB: low frequency head-to-back electrical stunning, HFHB: high frequency head-to-back electrical stunning. Values with different superscripts differ significantly at p<0.05. LW: live body weight pre-slaughter. Values are means ± 1 standard error (n = 8).

The results for blood loss in goats subjected to different slaughter methods are shown in Fig 2. Goats subjected to SWS (4.22%), LFHO (4.14%) and HFHB (4.09%) had higher (p<0.05) blood loss compared to those subjected to LFHB (2.82%). The main factors influencing bleeding are patency and size of the sticking wound, the blood vessels severed, cardiac arrest, muscle contractions, time (to bleed) carcass orientation and dressing procedures (to allow blood to escape) [31], which are all determined by the slaughter method. Greater blood loss during slaughtering without stunning (SWS) and slaughtering following recoverable electrical stunning techniques (LFHO and HFHB) could be due to the lack of cardiac arrest on applying the procedures. It has been reported that when animals are bled, about 75–85% of the total volume of blood in the body is lost when the heart is still beating [16,19], which is aproximately 4% of live weight. A beating heart is indispensable for a thorough bleed-out by the animal and attains higher exsanguination. The current finding is consistent with those of Kirton et al. [32] who observed that the lambs subjected to low frequency head-to-back electrical stunning had a lower blood loss compared with lambs subjected to head-only electrical stunning or slaughtered without stunning. The low bleeding efficiency in LFHB goats could be due to the incidence of ventricular fibrillation and stoppage of the heart at the time of current application as well as death of the animal immediately after stunning [33]. The blood loss in SWS and LFHO goats were similar (p<0.05). This observation concurs with those of Anil et al. [34] who observed similar blood loss in sheep subjected to pre-slaughter head-only electrical stunning and those slaughtered without stunning. A similar trend was reported by Khalid et al. [35] who indicated that there were no significant differences in blood loss at exsanguination in lambs slaughtered without stunning, subjected to head-only electrical stunning or post-cut head-only electrical stunning methods. Conversely, Velarde et al. [36] observed that the amount of blood loss in lambs subjected to head-only electrical stunning was higher compared with non-stunned lambs. The efficacy of pre-slaughter high frequency electrical currents to stun red meat animals has been reviewed by Farouk et al. [16,19]. The inability of high frequency electrical stun to cause cardiac arrest makes it acceptable in halal slaughter by some adherents of Islamic faith [16]. In addition, high frequency electrical stunning has been shown to enhance bleeding efficiency [31]. This factor is likely to affect bleeding efficiency by preventing any blood pressure rise, reducing the incidence of cardiac arrest and subsequently increasing the extent and rate of blood loss [37]. Mouchoniere et al. [38] reported that bleeding efficiency was higher and the incidence of cardiac arrest was lower with high frequency electrical stun. Similarly, Contreras and Beraquet [39] found higher blood loss when birds were subjected to high frequency of 1000 Hz compared to those subjected to lower frequencies. In the present study, the blood loss values were almost 1.5 times higher in HFHB than LFHB. The implication of this finding is that LFHO and HFHB could be applied in halal slaughter of fully conscious animals. The consumption of blood is forbidden in Islam and Jewish religion [16,17]. Thus, draining as much blood as possible out of the carcass is one of the major aims of religious slaughter. In addition, modern slaughter practices in Europe and North America require proper bleeding of slaughtered animals meant for food [9].

### Residual hemoglobin concentration

**Fig 3:**
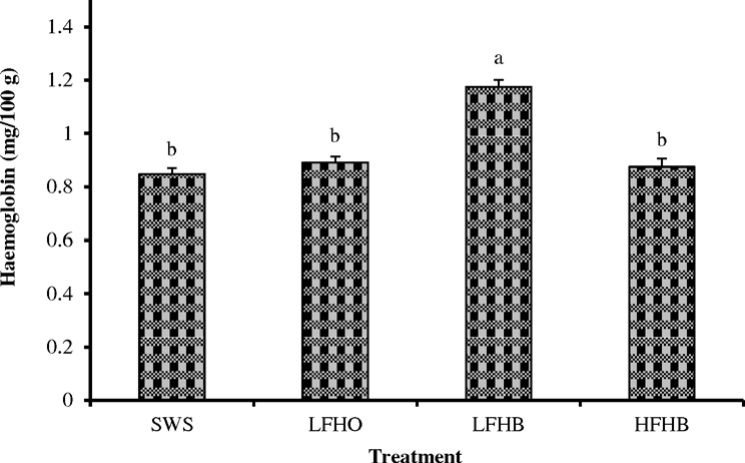
Hemoglobin content of *longissimus lumborum* muscle in goats subjected to slaughter without stunning and slaughter following different methods of electrical stunning. SWS: slaughter without stunning, LFHO: low frequency head-only, electrical stunning, LFHB: low frequency head-to-back electrical stunning, HFHB: high frequency head-to-back electrical stunning. Values with different superscripts differ significantly at p<0.05. Values are means ± 1 standard error (n = 8).

Fig 3 presents the residual hemoglobin concentration in LL muscle from goats subjected to different slaughter methods. The LFHB goats had higher (p<0.05) residual hemoglobin compared with SWS, LFHO and HFHB goats. This observation could be due to the lower blood loss in LFHB compared with SWS, LFHO and HFHB. Oellingrath et al. [40] showed that the meat hemoglobin content depends on the extent of carcass bleeding and the vascular bed in the muscles. The current findings are in agreement with those of Addeen et al. [10] and Alvarado et al. [14], who observed that broiler chickens subjected to pre-slaughter electrical stunning and those slaughtered without stunning had similar residual hemoglobin contents. It is worth mention that minimal anesthesia did not affect residual hemoglobin concentration in goats [11].

### Lipid oxidation

Table 1 shows the TBARS value of LL muscle from goats subjected to different slaughter methods. Regardless of slaughter method, the TBARS value increased (p<0.05) over storage. This observation is in agreement with those of Adeyemi et al. [41] and Nakyinsige et al. [27]. Slaughter method was not a source of variation influencing TBARS value in goat meat on d 0, 1, and 3 postmortem. Nonetheless, at 7 and 14 d postmortem, the LFHB meat had higher (p<0.05) TBARS value than meat from other slaughter techniques. This observation coincides with the results obtained for residual hemoglobin, which showed that LFHB had greater residual hemoglobin compared with SWS, LFHO and HFHB. Carcasses with residual blood increase the concentration of hemoglobin in meat [9]. Hemoglobin is a powerful promoter of lipid oxidation [14,42,43]. Hemoglobin have four polypeptide chains each comprising one heme group, with an iron atom harmonized within a porphyrin ring [44]. Impairment of the porphyrin ring during storage can cause breakdown of heme molecules and subsequently release of iron. The released of iron can stimulate lipid oxidation of muscle during the extended storage [10]. Apart from hemoglobin, the blood is also capable of generating superoxide, hydrogen peroxide and hydroxyl radicals, which are known to enhance the lipid oxidation [10,45]. This explains why low frequency head-to-back electrical stun, which results in low bleed-out, had greater lipid oxidation. The results obtained from this study show that lipid oxidation of meat from SWS was comparable to that from LFHO and HFHB. Similarly, Linares et al. [46] found no significant difference in lipid oxidation between head-only electrically stunned and non-stunned lambs. Lipid oxidation is involved in the deterioration of flavor, formation of rancid odors and discoloration [6]. Despite the increase in TBARS over storage, the range of TBARS value observed in the current study is less than 5 mg MDA-equivalents /kg meat, which is threshold value [4] for the detection of off-odors and off-taste for humans.

**Table 1:** Malondialdehyde equivalents content (mg/kg) of *longissimus lumborum* muscle during postmortem aging periods in goats subjected to slaughter without stunning and slaughter following different methods of electrical stunning (mean ± SE, n = 8).

Time postmortem (days)	Treatment			
	SWS	LFHO	LFHB	HFHB
**0**	0.483 ± 0.016^w^	0.487 ± 0.018^w^	0.493 ± 0.028^v^	0.490 ± 0.024^w^
**1**	0.597 ± 0.021^w^	0.616 ± 0.051^w^	0.696 ± 0.055^w^	0.600 ± 0.028^w^
**3**	0.798 ± 0.034^z^	0.813 ± 0.011^z^	0.906 ± 0.057^z^	0.806 ± 0.015^z^
**7**	1.380 ± 0.038^b^^,^^y^	1.430 ± 0.045^b^^,^^y^	1.941 ± 0.055^a^^,^^y^	1.412 ± 0.045^b^^,^^y^
**14**	2.492 ± 0.119^b^^,^^x^	2.585 ± 0.073^b^^,^^x^	3.148 ± 0.037^a^^,^^x^	2.509 ± 0.110^b^^,^^x^

SWS: slaughter without stunning, LFHO: low frequency head-only, electrical stunning, LFHB: low frequency head-to-back electrical stunning, HFHB: high frequency head-to-back electrical stunning.

^a,b^ means within the same row with different superscripts are significantly different (p<0.05).

^v,w,x,y,z^ means within the same column with different superscripts are significantly different (p<0.05).

### Meat protein oxidation

Protein oxidation is a major issue in meat quality assessment because muscle tissues involve high amounts of proteins that play significant roles in meat quality [6]. Meat protein oxidation is often evaluated by measuring the amount of protein thiols (the sulfhydryl group (SH) of a cysteine residue) [27] and carbonyl group [8,47].

#### Carbonyl content

Carbonylation is one of the most significant chemical modifications in oxidized proteins [8,48]. The carbonyl content of chevon obtained from goats subjected to different slaughter techniques is shown in Table 2. Slaughter method had no effect (p>0.05) on carbonyl content in goat meat. Regardless of slaughter method, the carbonyl content increased (p<0.05) over storage. These observations are consistent with the report of earlier trials [49–51], which showed that the amount of carbonyl groups significantly increased as meat ages. The range of carbonyl content is consistent with those of observed in broiler meat [52], chevon [53] and beef [54].

**Table 2:** Carbonyl content (nmol/mg protein) of *longissimus lumborum* muscle during postmortem aging periods in goats subjected to slaughter without stunning and slaughter following different methods of electrical stunning (mean ± SE, n = 8).

Time postmortem (days)	Treatment			
	SWS	LFHO	LFHB	HFHB
**0**	1.255 ± 0.132^x^	1.274 ± 0.063^w^	1.265 ± 0.059^w^	1.256 ± 0.073^w^
**1**	1.414 ± 0.111^x^	1.429 ± 0.094^x^	1.508 ± 0.0682^x^	1.421 ± 0.101^x^
**3**	1.878 ± 0.198^x^	2.036 ± 0.172^x^	2.371 ± 0.176^x^	2.110 ± 0.118^x^
**7**	3.2788 ± 0.256^y^	3.779 ± 0.307^y^	4.133 ± 0.111^y^	3.693 ± 0.352^y^
**14**	4.7061 ± 0.313^z^	5.241 ± 0.375^z^	6.602 ± 0.461^z^	5.091 ± 0.314^z^

SWS: slaughter without stunning, LFHO: low frequency head-only, electrical stunning, LFHB: low frequency head-to-back electrical stunning, HFHB: high frequency head-to-back electrical stunning.

^w,x,y,z^ means within the same column with different superscripts are significantly different (p<0.05).

#### Protein thiols

The results for the influence of slaughter methods on thiol content in chevon are presented in Table 3. The thiol concentration decreased from 43.9 to 32.2 nmol per mg protein, from 44.4 to 30.8 nmol per mg protein, from 44.6 to 28.9 nmol per mg protein and from 44.3 to 30.6 nmol per mg protein for SWS, LFHO and LFHB or HFHB, respectively. Thiol concentration was not influenced (p>0.05) by slaughter method. This observation concurs with that of Nakyinsige [55] in rabbits and Sabow et al. [21] in goats who found that the thiol concentration was not influenced by slaughter methods. The decrease in free thiol over storage is in tandem with previous reports on chill storage of meat [8,27,56]. The presence of transition metal ions by radical-mediated reactions may instigate the oxidation of thiol groups may be oxidized to produce thiyl radicals (RS•), which can react to form cross-linked structures [57]. Thiol groups from cysteine residues in biological systems are particularly susceptible to oxidation by almost all forms of reactive oxygen species [48]. It was observed that thiols only oxidized to a certain level, which means that thiol-containing cysteine residues in the myofibrillar proteins exhibit diverse reactivity [27]. This observation suggests that some of the thiol groups are hidden inside the core of the protein, and therefore protected from oxidation. The decrease in thiols corresponds to the oxidation of accessible thiol groups from cysteine residues that are located at the surface of the protein where as those from cysteine residues located in the core of the protein could be protected against free radical attack [58].

**Table 3:** Free thiol (SH) content (nmole/mg protein) of *longissimus lumborum* muscle during postmortem aging periods in goats subjected to slaughter without stunning and slaughter following different methods of electrical stunning (mean ± SE, n = 8).

Time postmortem (days)	Treatment			
	SWS	LFHO	LFHB	HFHB
**0**	43.9 ± 0.99^w^	44.4 ± 0.41^w^	44.6 ± 0.38^w^	44.3 ± 0.45^w^
**1**	42.9 ± 0.90^x^	43.9 ± 0.71^x^	43.0 ± 0.78^x^	43.5 ± 0.62^x^
**3**	40.2 ± 1.21^x^	39.8 ± 0.67^x^	38.9 ± 1.24^x^	39.7 ± 0.88^x^
**7**	36.3 ± 1.55^y^	34.5 ± 0.78^y^	33.7 ± 0.87^y^	34.2 ± 1.33^y^
**14**	32.2 ± 1.40^z^	30.7 ± 0.83^z^	28.9 ± 0.80^z^	30.6 ± 0.68^z^

SWS: slaughter without stunning, LFHO: low frequency head-only, electrical stunning, LFHB: low frequency head-to-back electrical stunning, HFHB: high frequency head-to-back electrical stunning.

^w,x,y,z^ means within the same column with different superscripts are significantly different (p<0.05).

### Myofibrillar protein profile

Electrophoresis was carried out in order to observe the modifications induced by slaughter methods and postmortem aging on myofibrillar proteins in goat meat. SDS-PAGE patterns showed a decline in the intensity of bands corresponding to myosin heavy chain (MHC) and troponin-T as postmortem days increased (Table 4). The actin band was relatively more stable. The reduction in band intensity is a sign of protein oxidation in muscle during chill storage. The current results are in line with those of Sabow et al. [56], Xue et al. [59] and Adeyemi et al. [60] which showed that increased protein oxidation enhance the degradation of myosin heavy chain and troponin-T but had no effect on the actin degradation. According to Marino et al. [61] and Bernevic et al. [62], the oxidation of myosin heavy chain occurs naturally in meat during aging. The intensity of MHC, troponin-T and actin proteins were quantified by measuring the reflective density (RD) of each detected band. As shown in Table 4, there was no variation in RD of MHC, troponin-T and actin with respect to slaughter method. The RD of MHC and troponin-T reduced significantly (p<0.05) as aging period increased while that of actin was stable during 14 d storage at 4°C. These observations concur with those of Sabow et al. [21] in goats and Nakyinsige [55] in rabbits who observed that slaughter methods had no effect on the reflective density of actin, troponin-T and myosin heavy chain while concentrations of myosin heavy chain and troponin-T decreased over storage. The similarity in degradation of myofibrillar proteins between the slaughter methods could be due to the similarity in protein oxidation as demonstrated through loss of thiol groups and increase in carbonyl concentrations.

**Table 4:** Reflective density/mm^2^ of myosin heavy chain, actin and troponin-T of *longissimus lumborum* muscle during postmortem aging periods in goats subjected to slaughter without stunning and slaughter following different methods of electrical stunning (mean ± SE, n = 8).

Time postmortem (days)	Treatment			
	SWS	LFHO	LFHB	HFHB
**Myosin heavy chain**				
**0**	65.981 ± 2.047^w^	65.065 ± 3.219^w^	65.343 ± 4.326^w^	64.559 ± 2.863^w^
**3**	61.653 ± 3.766^x^	60.132 ± 4.231^x^	59.889 ± 3.443^x^	60.373 ± 3.832^x^
**7**	54.059 ± 2.652^y^	52.472 ± 2.177^y^	50.267 ± 2.685^y^	51.909 ± 4.056^y^
**14**	47.815 ± 3.369^z^	45.342 ± 2.441^z^	43.732 ± 2.453^z^	45.668 ± 3.395^z^
**Actin**				
**0**	20.865 ± 0.581	20.963 ± 1.771	20.612 ± 1.096	20.685 ± 2.079
**3**	20.149 ± 1.345	20.196 ± 1.476	19.910 ± 1.157	20.083 ± 1.781
**7**	19.507 ± 1.394	19.406 ± 1.804	19.107 ± 1.286	19.991 ± 1.603
**14**	18.078 ± 1.442	18.983 ± 1.497	18.116 ± 1.313	18.207 ± 1.951
**Troponin-T**				
**0**	14.490 ± 0.563^w^	15.154 ± 1.239^w^	14.354 ± 0.578^w^	14.727 ± 0.852^w^
**3**	12.451 ± 0.831^x^	11.766 ± 0.954^x^	11.661 ± 1.162^x^	11.993 ± 0861^x^
**7**	9.875 ± 0.619^y^	8.454 ± 0.612^y^	8.653 ± 0.768^y^	10.488 ± 0.837^y^
**14**	6.901 ± 0.415^z^	6.474 ± 0.383^z^	5.653 ± 0.537^z^	6.881 ± 0.528^z^

SWS: slaughter without stunning, LFHO: low frequency head-only, electrical stunning, LFHB: low frequency head-to-back electrical stunning, HFHB: high frequency head-to-back electrical stunning.

^w,x,y,z^ means within the same column with different superscripts are significantly different (p<0.05).

Myosin is the most abundant protein in the myofibril and contributes to the structure and tensile strength of meat [61]. Electrophoretic analysis studies by Sun et al. [58] and Addeen et al. [10] observed that the protein band corresponding to MHC was the most sensitive protein to oxidation, whereas lower molecular weight proteins seemed to oxidize later. Ooizumi and Xiong [63] showed that the initial oxidation of chicken myofibrils led to changes in myosin, especially the modifications of thiol groups at the myosin ATPase active site and intermolecular cross-linking of MHC. According to Xue et al. [59] and Morzel et al. [26], highly oxidative conditions cause cross-linking, polymerization and aggregate formation in MHC through disulfide bonds, bityrosine and carbonyl. These cross-linkages and aggregates are more resistant to enzymatic degradation compared to native protein forms [64]. The decrease of the band corresponding to myosin heavy chains could be due to the enzymatic degradation during meat ripening.

Actin can assemble into various structures and partakes in various processes in eukaryotic cells, and has been reported to be μ-calpain substrate [65]. It has been reported that actin and actin-bundling proteins play a significant role in muscle contraction [59]. The present research showed that actin was not affected by both slaughter method and postmortem storage at 4°C. This oxidative stability of actin may be attributable to inaccessibility of oxidation sites, in which myofibrillar suspensions may be masked by the interaction of actin with myosin chains [59]. Bandman and Zdanis [66] indicated that actin is degraded very little or not at all during meat aging at 0–5°C, even after 56 days.

Troponin-T is the tropomyosin-binding component of the troponin complex that is involved in the calcium-dependent regulation of skeletal muscle contraction [67]. It is present in the I-band regions of the intact myofibril, which undergo considerable breakage during muscle aging [67]. Troponin-T band intensity reduced over postmortem storage. This observation is in tandem with those of Martinaud et al. [54]. Santé-Lhoutellier et al. [49] found that the degradation of troponin-T of lamb muscles was affected during the postmortem storage. However, Gadiyaram et al. [68] reported that aging of goat meat for 4 days did not significantly affect the major myofibrillar proteins.

### Microbiological status

**Fig 4:**
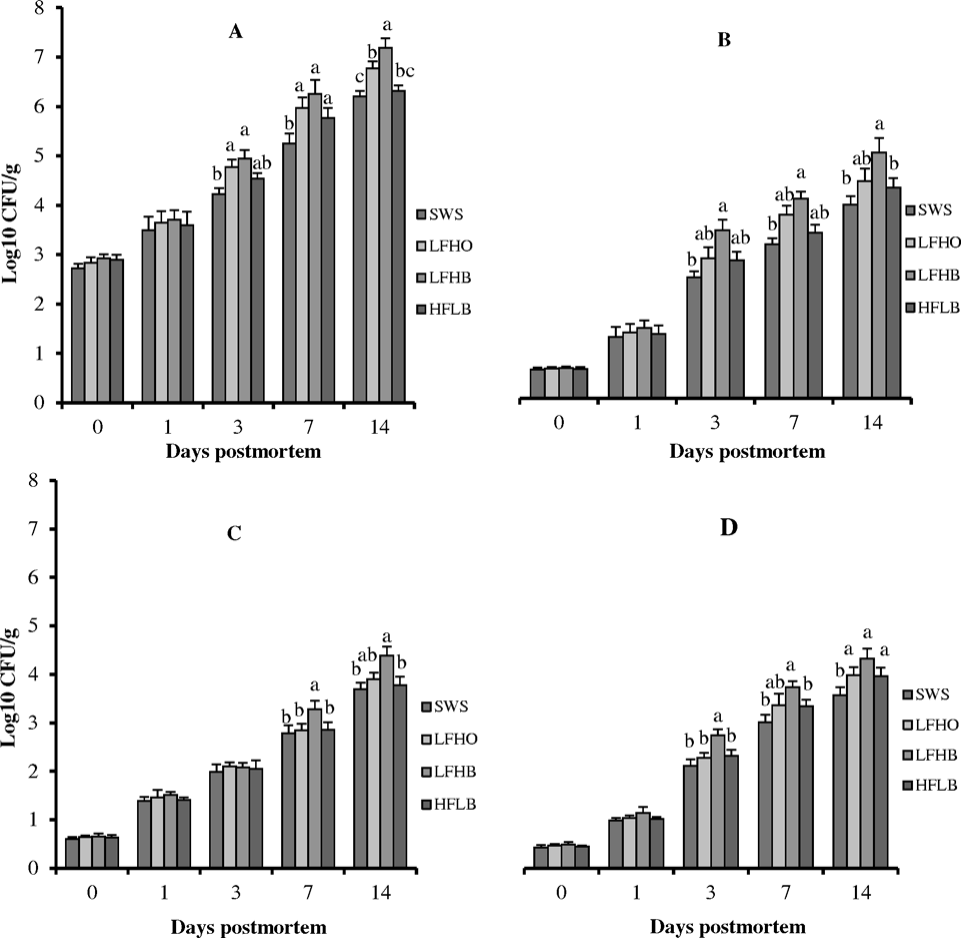
Microbiological quality during postmortem aging periods of *semitendinosus* muscle in goat subjected to slaughter without stunning and slaughter following different methods of electrical stunning. Graphs A-D: the population of total aerobic counts, *Pseudomonas* spp., lactic acid bacteria and *Enterobacteriaceae*, respectively. SWS: slaughter without stunning, LFHO: low frequency head-only, electrical stunning, LFHB: low frequency head-to-back electrical stunning, HFHB: high frequency head-to-back electrical stunning. Values within day with different superscripts differ significantly at p<0.05. Values are means ± 1 standard error (n = 8).

Microbiological proliferation in meat is affected by various factors such as the physiological status of the animal during slaughter time, the spread of microbes during slaughter and storage conditions [69]. It is noteworthy that all animals in this study were subjected to minimal anesthesia even though prior findings have shown that minimal anesthesia had negligible effect on microbial analysis [11]. The impacts of slaughter method on microbiological status of chevon during a 14 d postmortem refrigerated storage are presented in Fig 4. The microbial counts in chevon at d 0 and 1 were not influenced (p>0.05) by slaughter method. Nonetheless, at d 3 postmortem, a higher (p<0.05) total aerobic counts, *Pseudomonas* spp. and *Enterobacteriaceae* counts were present in meat samples obtained from the LFHB group. Similarly, the aerobic count of both the LFHO and LFHB is higher compared to the SWS sample at d 3. The population of lactic acid bacteria was not affected (p>0.05) by slaughter method at d 3. At d 7 postmortem, meat samples obtained from the SWS exhibited significantly lower total aerobic counts than the stunning groups. However, samples from LFHB had the highest (p<0.05) counts of *Pseudomonas* spp., *Enterobacteriaceae* and lactic acid bacteria compared to SWS, LFHO and HFHB after both 7 and 14 d. At 14 days of storage at 4°C, meat from the LFHB and LFHO animals showed significantly higher levels of total aerobic counts than that of the SWS and HFHB groups, while the *Enterobacteriaceae* count of all the stunning groups (LFHO, LFHB and HFHB) was higher compared to SWS group. Generally, the growth of microorganisms increased as postmortem storage progressed in meat samples from all slaughter groups. However, the microbial growth was more prominent in electrically stunned groups compared to the SWS group. This observation could be due to the faster postmortem pH decline of meat from electrical stunned goats (5.98 × 10^−2^, 5.79 × 10^−2^ and 5.58 × 10^−2^ for LFHO, LFHB and HFHB, respectively) compared to SWS goats (3.43 × 10^−2^). A similar explanation was given by Bórnez et al. [1] who attributed the higher bacterial growth exhibited by the electrically stunned group over storage to significantly faster pH decline which causes aging to start earlier in the lamb meat. Additionally, the greater bacterial counts in LFHB samples could be due to the lower blood loss observed in the group. According to Nakyinsige et al. [9], the amount of blood left within the carcass after bleeding is one of the major factors affecting the extent of contamination and the degree of spoilage. Blood favors multiplication of spoilage microorganisms [70]. The results of the present study show that the levels of all microbes detected throughout the 14 days storage in meat from SWS were lower as compared to other methods of slaughtering (LFHO, LFHB and HFHB). These observations corroborate the findings of Sabow et al. [11] in goats, Nakyinsige et al. [9] in rabbits and Addeen et al. [10] and Ali et al. [71] in broiler chicken who found lower counts of bacteria in meat obtained from animals slaughtered without stunning.

## Conclusion

The results from the present study indicate that blood loss, and lipid and protein oxidation as well as microbiological quality of meat from goats subjected to slaughter without stunning is comparable to that from electrically stunned prior slaughter with low frequency head-only and high frequency head-to-back. However, low frequency head-to-back electrical stunning method had detrimental effects on the shelf life of goat meat due to its low bleed-out. High frequency electrical stunning can be used as a substitute for conventional electrical stunning techniques to yield goat meat with better shelf life during postmortem aging.
