# Correction: Bleeding Efficiency, Microbiological Quality and Oxidative Stability of Meat from Goats Subjected to Slaughter without Stunning in Comparison with Different Methods of Pre-Slaughter Electrical Stunning

**DOI:** 10.1371/journal.pone.0178890

**Published:** 2017-05-26

**Authors:** The PLOS ONE Staff

The corresponding author is listed incorrectly. The correct corresponding author is Awis Qurni Sazili. The publisher apologizes for the error.
